# Hypercarbia in a Pediatric Patient With Relapsed Medulloblastoma

**DOI:** 10.7759/cureus.30034

**Published:** 2022-10-07

**Authors:** Mitchell A Luangrath, Mariko Sato, Cody R Tigges

**Affiliations:** 1 Pediatric Critical Care Medicine, University of Iowa, Iowa City, USA; 2 Pediatric Oncology, Children's Hospital of Orange County, Orange, USA

**Keywords:** hypercarbic respiratory failure, pediatric oncology, pediatric critical care, medulloblastoma, pediatric respiratory failure

## Abstract

Pediatric medulloblastoma is a common form of pediatric brain tumor and typically presents with progressive signs of increased intracranial pressure and ataxia. Relapse of the disease is most often diagnosed on surveillance imaging. We present the case of a 13-year-old boy with a previous history of medulloblastoma who presented with chronic hypercarbic respiratory failure as a symptom of a recurrent tumor. Imaging demonstrated a left cerebellar enhancing mass with leptomeningeal thickness and extension to the posterior medulla oblongata, which is the center for respiratory control. His hypercarbic respiratory failure represents a unique presentation of a central nervous system (CNS) tumor. Thus, this case illustrates the importance of thorough evaluation for CNS tumors involving the brainstem in patients with respiratory acidosis and no clear pulmonary etiology.

## Introduction

Medulloblastoma accounts for 15%-20% of pediatric brain tumors with peak incidences at ages 3-4 and 8-10 years [1,2]. Long-term survival in patients with high-risk medulloblastoma is approximately 70% [2]. Recurrent disease occurs in 31.2% of patients with a five-year survival of 12.4% [3]. Initially, medulloblastoma most commonly presents with signs of increased intracranial pressure and cerebellar dysfunction while most cases of recurrent medulloblastoma are diagnosed on surveillance imaging [2,4]. Respiratory compromise is not commonly identified on initial presentation with medulloblastoma or relapsed disease. Here, we present a case of a 13-year-old patient with a history of metastatic medulloblastoma who presented with increasing neurologic deficits and evidence of central hypoventilation eight years after the completion of chemotherapy and radiation therapy.

## Case presentation

The 13-year-old boy was initially diagnosed with metastatic medulloblastoma at 4 years of age after presenting with a one-month history of progressive headaches, nausea, and vomiting. Brain imaging demonstrated obstructive hydrocephalus secondary to multiple masses in the posterior fossa with a predominant mass in the cerebellum. He underwent posterior fossa craniotomy with resection of the left superior cerebellar and midline cerebellar vermis tumors. Pathology of the tumor demonstrated World Health Organization (WHO) grade IV desmoplastic variant medulloblastoma. Post-operatively, the patient had significant neurologic deficits and mental status alteration due to posterior fossa syndrome. An initial trial of extubation was unsuccessful due to apnea and concerns for airway protection, and he was ultimately able to be extubated following ventriculoperitoneal (VP) shunt placement.

**Figure 1:**
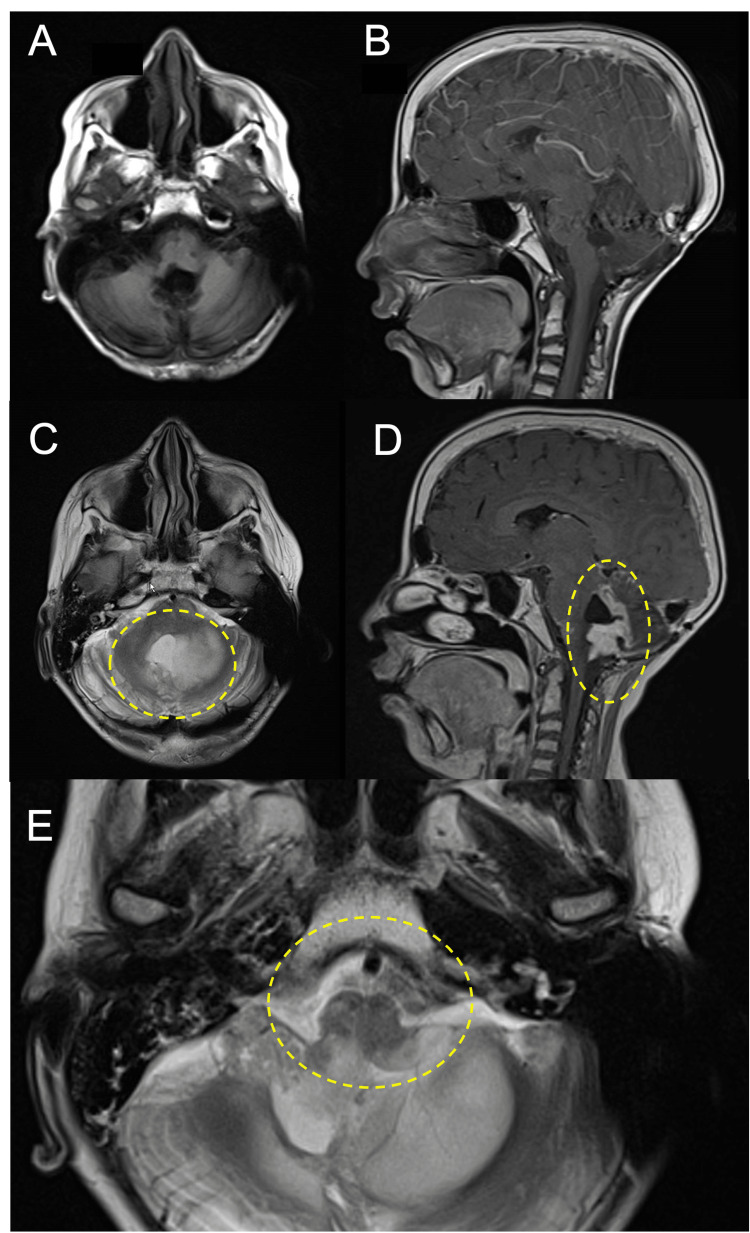
Comparative MRI imaging of the brain in a patient with medulloblastoma. a.) Axial and b.) sagittal gadolinium contrast images five years and eight months following completion of therapy without evidence of recurrence. c.) Axial and d.) sagittal gadolinium contrast images at the time of relapsed medulloblastoma showing enhancing nodular relapsed tumor extending from the fourth ventricle into the left cerebellum. There is extensive leptomeningeal enhancement extending from the fourth ventricle to the right cerebellopontine angle, into the right internal auditory canal, and along the right tentorium as well as enhancement along the right anterior medulla and the midbrain bilaterally. e.) Enlarged axial T2 sequence demonstrating posterior medulla involvement of the recurrent tumor.

The patient received craniospinal irradiation and adjuvant chemotherapy, which he completed about one year following his diagnosis. He had persistent neurologic deficits following treatment including right hemiparesis and facial nerve palsy. He also developed hearing loss, endocrinopathy, and neurocognitive deficits secondary to his therapy. The patient was followed every three months for two years, then biannually for two years, and then annually in the pediatric oncology clinic as is typically recommended [2]. During this time, he had no evidence of tumor recurrence on brain and spine magnetic resonance imaging (MRI) up to five years and eight months following treatment completion (Figure 1). 

Just over eight years following the completion of therapy, he presented to the emergency department with a one-month history of increasing somnolence, headaches, nausea, and worsening ataxia. On presentation, the patient was found to have oxygen desaturation to 86% in room air, which improved with the initiation of a high-flow nasal cannula (HFNC). The remainder of his vital signs on presentation were a heart rate of 88 beats per minute, blood pressure of 120/86 mmHg, respiratory rate of 14 breaths per minute, and temperature of 36°C. His neurologic examination was notable for right-sided weakness with 4/5 strength in the upper and lower extremities and right facial droop, which were stable compared to previous exams. While somnolent at times, he followed commands and answered questions appropriately. From a respiratory perspective, he had clear lungs to auscultation and normal work of breathing.

A venous blood gas was notable for a respiratory acidosis with pH 7.27, pCO2 79, pO2 38, and base excess of 11. There was no history of respiratory symptoms such as cough, nasal congestion, or dyspnea. His white blood cell count was 4,100/µL (4.1 x 109/L) (reference range (RR) 4500 - 13000/µL (4.5 - 13 x 109/L)) with normal hemoglobin and platelet count. The differential was within normal limits save for a marginally decreased lymphocyte count of 1230/µL (1.23 x 109/L) (RR 1250 - 7000/µL (1.25 - 7 x 109/L)). Electrolytes were within normal limits as well. C-reactive protein was normal at less than 0.5 mg/dL (< 5 mg/L). Chest and VP shunt series X-rays were unremarkable. Computed tomography (CT) scan of the brain demonstrated stable size and configuration of the ventricular system without any acute intracranial changes.

Given the lack of infectious symptoms and normal chest X-ray, infection including pneumonia or other primary pulmonary process were considered unlikely to explain the patient’s progressive neurologic changes and respiratory acidosis. Rather, his respiratory acidosis was felt to be central in nature and likely chronic given the metabolic compensation. The HFNC support was rapidly weaned to a conventional nasal cannula with no change in his respiratory status, oxygen saturation, or blood gas parameters. Further neurologic work-up was conducted with an electroencephalogram (EEG), which showed no seizure activity. MRI of the brain and spinal cord demonstrated an enhancing cerebellar mass at the surgical site with leptomeningeal thickening and enhancement along the fourth ventricle into the posterior fossa and up to the tentorium and midbrain (as seen in Figures 1C-1E) as well as diffuse leptomeningeal enhancement of the whole spine. These findings were considered consistent with recurrent medulloblastoma.

The family discussed options for treatment of the patient’s recurrent disease; however, given the extent of the disease burden and lack of curative therapy options, they elected to take their son home with palliative care. He passed away five weeks following discharge to home. 

## Discussion

Here, we present a rare presentation of relapsed medulloblastoma in a patient with chronic respiratory failure with hypercarbia and metabolic compensation along with hypoxemia. This was noted to occur in the absence of any other symptom that would suggest a primary respiratory or infectious etiology. The respiratory center is located in the pons and medulla of the brainstem. In particular, the medullary respiratory center, which is critical in directing the pattern of ventilation, is located just beneath the floor of the fourth ventricle [5]. MRI of the brain in this patient revealed an enhancing nodular mass and extensive leptomeningeal enhancement along the pons and medulla. The location of his disease recurrence likely explains his impaired ventilation and subsequent chronic respiratory acidosis.

This patient also failed tracheal extubation following his initial tumor resection surgery secondary to apnea and concerns for airway protection. At the time, it was felt that his need for reintubation was secondary to brainstem dysfunction. It has been reported that nearly 5% of adult patients require reintubation following craniotomy, most commonly due to neurologic deterioration [6]. Tracheal reintubation following craniotomy in pediatric patients is relatively rare with a reported rate of 0.94%; however, odds of reintubation are higher in patients 1-5 years of age and following procedures involving the posterior fossa [7]. Following VP shunt placement, our patient was successfully extubated. He showed no signs of impaired ventilation at any other time during treatment or remission, suggesting that his chronic respiratory failure and hypercarbia on re-presentation were indicators of tumor recurrence.

There are no reported cases in the literature of pediatric patients with respiratory acidosis and hypercarbia secondary to medulloblastoma, but impairments of ventilation have been demonstrated in cases of other central nervous system (CNS) tumors. In one such case, a 3-year-old patient with central hypoventilation syndrome secondary to pilocytic astrocytoma involving the fourth ventricle and extending to the medulla required tracheostomy placement and showed gradual improvement in ventilation after surgical resection but still required mechanical ventilation via tracheostomy overnight [8]. Another case report describes a 4-year-old with a ganglioma involving the cerebellum and pons who presented with hypercarbia, hypoxemia, and periodic breathing during sleep [9]. In these cases, there is tumor involvement of the brainstem and central respiratory centers leading to alterations in ventilation and respiratory control. As each case represents a unique neoplasm, it suggests that the location rather than the type of malignancy has a greater impact on respiratory impairment.

Our patient had imaging without signs of recurrent disease nearly six years following the completion of treatment, and at the time of re-presentation with recurrent medulloblastoma, over eight years had passed since the completion of his therapy for medulloblastoma. Following completion of treatment, surveillance evaluations with radiographic exams occur in decreasing frequency one year at a time beginning with follow-up every three months, then every 3-4 months, then every six months, and then annually [2]. A more recent study of relapsed medulloblastoma revealed that patients who received craniospinal radiation upfront had a significantly prolonged time to relapse compared to patients who did not receive radiation at the start [10]. This result may support the extension of surveillance follow-up in patients with medulloblastoma, especially as the diagnosis of recurrent medulloblastoma on surveillance imaging is associated with increased survival [11]. In our case, the patient was initially followed more closely than typical guidelines would recommend; however, the decreased frequency of surveillance after the first few years could have contributed to the life-threatening presentation of his relapsed disease. 

## Conclusions

Here, we have presented the case of a pediatric patient with respiratory acidosis and progressive neurologic deficits secondary to recurrent medulloblastoma. As there are few reports in the literature of patients presenting with hypercarbia and chronic respiratory acidosis as a manifestation of a CNS tumor, this represents a unique presentation of relapsed metastatic medulloblastoma. Therefore, regular assessment of the respiratory status of patients with CNS neoplasms with pulse oximetry or blood gases may prove useful. Furthermore, consideration should be made about extending the duration of regular follow-up to allow for an earlier diagnosis of relapsed disease. Finally, this case illustrates the importance of considering CNS pathologies in patients presenting with impairments of oxygenation or ventilation without clear pulmonary etiologies.
